# Supplementary material for: Quantum wave mixing and visualisation of coherent and superposed photonic states in a waveguide
Source: Nat Commun. 2017 Nov 7;8:1352. doi: 10.1038/s41467-017-01471-x (PMC5676721; doi:10.1038/s41467-017-01471-x)
Supplement: Supplementary file 1 — Supplementary Information [file 41467_2017_1471_MOESM1_ESM.pdf]

Supplementary Note 1. Elastic scattering of electromagnetic waves on an artificial atom

A two-level atom with ground and excited states $|g\rangle$ and $|e\rangle$ and energy splitting $\hbar\omega_0$, interacting with a quantised field at frequency ω can be described by the interaction picture Hamiltonian

$$H = i\hbar g(a^\dagger \sigma^- e^{i\delta\omega t} - a\sigma^+ e^{-i\delta\omega t}), \tag{1}$$

where $\hbar g$ is the coupling energy, $\delta\omega = \omega - \omega_0$ is small detuning, $\sigma^+ = |e\rangle\langle g|$ ($\sigma^- = |g\rangle\langle e|$) is the rising (lowering) operator of the atomic states and $a^\dagger$ (a) is the creation (annihilation) operator of photon states $|n\rangle$ at frequency $\omega = \omega_0 + \delta\omega$ (n is an integer number larger or equal than zero). An evolution of the system on a short time interval $\Delta t \ll \delta\omega^{-1}$ is described by operator $U(t', t) = \exp(-\frac{i}{\hbar}H_t\Delta t)$, where H_t is the Hamiltonian at time t, $\Delta t = t' - t$. It can be expanded into an infinite series according to

$$U(t', t) = 1 + \eta(a^\dagger s^- - as^+) - \frac{\eta^2}{2!}(aa^\dagger s_e + a^\dagger a s_g) - \frac{\eta^3}{3!}(a^\dagger a a^\dagger s^- - aa^\dagger a s^+) + ..., \tag{2}$$

where $\eta = g\Delta t$ and $s^\pm$ are time dependent operators $s^+(t) = \sigma^+ e^{-i\delta\omega t}$ and $s^-(t) = \sigma^- e^{i\delta\omega t}$. The evolution operator can be simplified to

$$U(t', t) = \cos\left(\eta\sqrt{a^\dagger a}\right)s_g + \cos\left(\eta\sqrt{aa^\dagger}\right)s_e - \frac{as^+}{\sqrt{a^\dagger a}}\sin\left(\eta\sqrt{a^\dagger a}\right) + \frac{a^\dagger s^-}{\sqrt{aa^\dagger}}\sin\left(\eta\sqrt{aa^\dagger}\right), \tag{3}$$

where $s_e = s^+s^-$ and $s_g = s^-s^+$ and can be further rewritten as

$$U(t', t) = \sum_{n=0}^{\infty}\Big[\cos\left(\eta\sqrt{n}\right)|n\rangle\langle n|s_g + \cos\left(\eta\sqrt{n+1}\right)|n\rangle\langle n|s_e - \\ |n-1\rangle\langle n|s^+\sin\left(\eta\sqrt{n}\right) + |n+1\rangle\langle n|s^-\sin\left(\eta\sqrt{n+1}\right)\Big]. \tag{4}$$

Particularly for initial state $\Psi(t) = |g, n\rangle$, the evolution results in $\Psi(t') = \cos(\eta\sqrt{n})|g, n\rangle - e^{-i\delta\omega t}\sin(\eta\sqrt{n})|e, n-1\rangle$. We consider the evolution under a drive of strong coherent radiation $|\alpha\rangle$, where real $\alpha \gg 1$. When $\Psi(t) = |g, \alpha\rangle$, the evolution is simplified to

$$\Psi(t') \approx \cos\frac{\theta}{2}|g, \alpha\rangle - e^{-i\delta\omega t}\sin\frac{\theta}{2}|e, \alpha'\rangle, \tag{5}$$

where $\theta = 2\eta\alpha$, $\alpha = \sqrt{\langle n\rangle}$ and $|\alpha'\rangle = \left(1 - e^{-|\alpha|^2}\right)^{-1/2}\sum_{n=1}^{\infty}|n-1\rangle\langle n|\alpha\rangle$. The interesting for us case is $\alpha \gg 1$ and therefore one photon absorption does not effectively change the state: $\alpha' \approx \alpha$. We can rewrite the state in Supplementary Eq. (5) as $\Psi(t') \approx (\cos\frac{\theta}{2}|g\rangle - e^{-i\delta\omega t}\sin\frac{\theta}{2}|e\rangle) \otimes |\alpha\rangle$. After switching off the pulse, the photon states of the driving field collapse to zero ($|\alpha\rangle \to |0\rangle$) and the system state becomes

$$\Psi' = \left(\cos\frac{\theta}{2}|g\rangle - e^{-i\delta\omega t}\sin\frac{\theta}{2}|e\rangle\right) \otimes |0\rangle \tag{6}$$

and

$$\langle s^+\rangle = -\frac{\sin\theta}{2}. \tag{7}$$

The superposed system (at $\theta \neq M\pi$, where M is an integer number) acquires phase $\delta\omega t$ from the incident coherent wave [1, 2] and then generates a superposed single-photon state. It is instructive to analyse the evolution of Ψ' (from Supplementary Eq. (6)) under the operator from Supplementary Eq. (4). When the accumulated angle $\eta = \frac{\pi}{2}$,

$$U_{ap} = |0\rangle\langle 0|\sigma^-\sigma^+ + e^{i\delta\omega t}|1\rangle\langle 0|\sigma^- \tag{8}$$

and the atomic superposition is converted into the superposition of a single-photon field at frequency ω according to

$$U_{ap}\left[\left(\cos\frac{\theta}{2}|g\rangle - e^{-i\delta\omega t}\sin\frac{\theta}{2}|e\rangle\right) \otimes |0\rangle\right] = |g\rangle \otimes \left(\cos\frac{\theta}{2}|0\rangle - \sin\frac{\theta}{2}|1\rangle\right). \tag{9}$$

We introduce single-photon creation operator $b^+ = |1\rangle\langle 0|$ at frequency ω and then

$$\langle b^+\rangle = -\frac{1}{2}\sin\theta. \tag{10}$$

Supplementary Eqs. (7 - 10) can now be rewritten using b-operators and the important consequence is that the atomic superposition is converted into the coherent single-photon field by substitution $s^+ \to b^+$. In the more general case, the atomic coherence is mapped on the zero-one photon state according to $s^+ \to b^+$ and $s^- \to b^-$, where $b^- = |0\rangle\langle 1|$.

The classical coherent and zero-one states can be repesented in similar forms

$$|\alpha\rangle = A\Big(|0\rangle + \alpha|1\rangle + \frac{\alpha^2}{\sqrt{2!}} + ...\Big) \tag{11a}$$

$$|\beta\rangle = B\big(|0\rangle + \beta|1\rangle\big), \tag{11b}$$

where $A = \exp(-|\alpha|^2/2)$ and $B = (1+|\beta|^2)^{-1/2}$. Particularly for the coherent photon state in Supplementary Eq. (9), $\beta = -\tan\theta/2$ and $B = \cos\theta/2$.

The Hamiltonian of Supplementary Eq. (1) can be equivalently rewritten through the single-photon creation/annihilation operators as

$$H' = i\hbar g(b^+a - b^-a^\dagger), \tag{12}$$

meaning that b-operators describe atomic excitations with phase $\delta\omega t$ and, therefore, satisfy identities similar to s-operators: $b^+b^- = |1\rangle\langle 1|$, $b^-b^+ = |0\rangle\langle 0|$, $b^+b^+ = 0$, $b^-b^- = 0$.

We can simplify Supplementary Eq. (4) for the case of the strong coherent wave drive as

$$U(t', t) \approx \cos\big(\eta\sqrt{a^\dagger a}\big) + (a^\dagger b^- - ab^+)\frac{\sin\big(\eta\sqrt{a^\dagger a}\big)}{\sqrt{a^\dagger a}}. \tag{13}$$

Supplementary Note 2. A two-level atom driven by two microwaves

Now we will discuss an atom driven by two different frequency waves ($\omega_\pm = \omega_0 \pm \delta\omega$). For this case we can calculate the spectra within our semiclassical approach, using Supplementary Eqs. (5, 6). Substituting classical driving field $\theta = \Omega e^{i\delta\omega t} + \Omega\Delta t e^{-i\delta\omega t} = 2\Omega\Delta t\cos\delta\omega t$ into Supplementary Eq. (7), we obtain

$$\langle s^+\rangle = -\frac{1}{2}\sin(2\Omega\Delta t\cos\delta\omega t), \tag{14}$$

which is decoupled in Bessel function series according to

$$\langle s^+\rangle = -\sum_{k=-\infty}^{\infty}(-1)^k J_{2k+1}(2\Omega\Delta t)\cos[(2k+1)\delta\omega t], \tag{15}$$

and, therefore, calculation the spectral component $\langle s^+_{2k+1}\rangle$ of the emission at frequency $(2k+1)\delta\omega t$ gives:

$$\langle s^+_{2k+1}\rangle = \frac{(-1)^k}{2}J_{2k+1}(2\Omega t)e^{i(2k+1)\delta\omega t} \tag{16}$$

However, in spite of the simplicity of the derivation above, it does not illustrate the physical entity of QWM, in particular, does not seperate individual contributions of multi-photon processes into emission and the connection with photon statistics of the emitted light. Moreover, the semiclassical picture fails to explain the limited number of spectral components we observe for the case of quantum mixing with delayed pulses. Therefore, for our purposes it is instructive to calculate the same physical quantity from an approach of second quantization.

For this approach, we now take into account the two driving continuous coherent fields $|\alpha_-\rangle_-$ and $|\alpha_+\rangle_+$, where $\alpha_\pm$ are real amplitudes for simplicity. The Hamiltonian is then modified to

$$H_2 = i\hbar g(s^-_- a^\dagger_- - s^+_- a_- + s^-_+ a^\dagger_+ + s^+_+ a_+), \tag{17}$$

where $a^\dagger_\pm$ ($a_\pm$) is the creation (annihilation) operator of a photon at $\omega_\pm$, $s^\pm_+ = \sigma e^{\mp i\delta\omega t}$, $s^\pm_- = \sigma e^{\pm i\delta\omega t}$ and $\hbar g$ is the coupling energy to the modes. The evolution operator of the Hamiltonian of Supplementary Eq. (17) can be expanded

similarly to the Supplementary Eq. (4), however, each term contains sequential combinations of operators $s_\pm^- a_\pm^\dagger$ and $s_\pm^+ a_\pm$. We can rewrite the Hamiltonian through the b-operators, using substitution $s_\pm^+ \to b_\pm^+$ and $s_\pm^- \to b_\pm^-$,

$$H = i\hbar g\big(b_-^+ a_- - b_-^- a_-^\dagger + b_+^+ a_+ - b_+^- a_+^\dagger\big), \tag{18}$$

where $b_\pm^\pm$ describe atomic excitation/relaxation with phases $\pm\delta\omega t$. The evolution operator $U(t',t) = \exp(-\frac{i}{\hbar}H_t\Delta t)$ can be rewritten in the tensor form

$$U = 1 + \eta(b_m^- a_m^\dagger - b_m^+ a_m) - \frac{\eta^2}{2!}(b_m^+ b_j^- a_m a_j^\dagger + b_m^- b_j^+ a_m^\dagger a_j) - \frac{\eta^3}{3!}(b_{m-j+p}^- a_m^\dagger a_j a_p^\dagger - b_{m-j+p}^+ a_m a_j^\dagger a_p) + ..., \tag{19}$$

where indexes take values ± 1. We relay here on $b_m^+ b_j^- b_p^+ = b_{m-j+p}^+$ because b-operators should satisfy the same as s-operator relations: $s_m^+ s_j^- s_p^+ = e^{-im\delta\omega t}\sigma^+ e^{ij\delta\omega t}\sigma^- e^{-ip\delta\omega t}\sigma^+ = e^{-i(m-j+p)\delta\omega t}\sigma^+ = s_{m-j+p}^+$. Here we expanded the definition of s-operators to an arbitrary l-mode according with $s_l^\pm = e^{\mp il\delta\omega t}\sigma^\pm$. This, for example, means that the third order terms $a_+ a_-^\dagger a_+ b_3^+$ and $a_- a_+^\dagger a_- b_{-3}^+$ result in creation of the single-photon fields at frequency $\omega_{\pm 3} = \omega_0 \pm 3\delta\omega$. More generally, the output light could be generated at frequencies $\omega_{\pm l} = \omega_0 \pm l\delta\omega$, where $l = 2k+1, k = 0, 1, 2, ...$ Among all terms in Supplementary Eq. (19) contributing into creation of the single-photon field at $\omega_{\pm l}$, the one of lowest order consists of $2k+2$ operators: $2k+1$ a-operators $a_\pm a_\mp^\dagger a_\pm ... = (a_\pm a_\mp^\dagger)^k a_\pm$ and one $b_{\pm(2k+1)}^+$.

As it was shown in [1], the superposed atom generates a coherent field $V = \frac{\hbar\Gamma_1}{\mu}\langle s^+\rangle$. Generalising the statement, we can write the expression for the single-photon coherent field generated at frequency $\omega_{\pm l}$:

$$V_{\pm l} = \frac{\hbar\Gamma_1}{\mu}\langle b_{\pm l}^+\rangle, \tag{20}$$

where μ is the dipole coupling moment for our case of capacitive coupling of the atom to the transmission line.

In order to analyze the evolution, we start from initial state $\Psi(t) = |\beta\rangle \otimes |n\rangle_- \otimes |n\rangle_+$ consisting of the atom in the superposition described by single-photon coherent state $|\beta\rangle$, and photon states $|n\rangle_\pm$ (where $n \gg 1$) with equal number of photons in both frequency modes. We introduce the following operators

$$\hat{A}_{2k}^{+-} = \begin{cases} (a_-^\dagger a_+)^{-k} & : k < 0 \\ (a_+^\dagger a_-)^k & : k \geq 0 \end{cases} \quad \hat{A}_{2k+1}^{-} = \begin{cases} (a_- a_+^\dagger)^{-k} a_- & : k < 0 \\ (a_+ a_-^\dagger)^k a_+ & : k \geq 0 \end{cases} \tag{21a}$$

$$\hat{A}_{2k}^{-+} = \begin{cases} (a_- a_+^\dagger)^{-k} & : k < 0 \\ (a_+ a_-^\dagger)^k & : k \geq 0 \end{cases} \quad \hat{A}_{2k+1}^{+} = \begin{cases} (a_-^\dagger a_+)^{-k} a_-^\dagger & : k < 0 \\ (a_+^\dagger a_-)^k a_+^\dagger & : k \geq 0, \end{cases} \tag{21b}$$

which satisfy relations $(\hat{A}^+)_{2k+1}^\dagger = \hat{A}_{2k+1}^-$, $(\hat{A}_{2k}^{+-})^\dagger = \hat{A}_{-2k}^{+-}$, $(\hat{A}_{2k}^{-+})^\dagger = \hat{A}_{-2k}^{-+}$. The operator

$$\hat{A}_{2k+1}^- b_{2k+1}^+ \tag{22}$$

creates a single photon at ω_{2k+1} with the least number of photons created/annihilated at driving frequencies $\omega_\pm$. Particularly, $A_{2k+1}^-|n_-, n_+\rangle = \big(\frac{(n_-+k)!}{n_-!}\frac{n_+!}{(n_+-k-1)!}\big)^{\frac{1}{2}}|n_- + k, n_+ - k - 1\rangle$, when $k > 0$. In the discussed case of large and equal photon number $n \gg 2k+1$, $A_{2k+1}^-|n, n\rangle \approx n^{k+\frac{1}{2}}|n+k, n-k-1\rangle$. The evolution can be simplified to

$$U(t',t)\Psi(t) \approx \sum_{k=-\infty}^{\infty}\left[\hat{A}_{2k}^{+-}C_{2k}^{+-}b_k^- b_{-k}^+ + \hat{A}_{2k}^{-+}C_{2k}^{-+}b_k^+ b_{-k}^- - \hat{A}_{2k+1}^- C_{2k+1}^- b_{2k+1}^+ + \hat{A}_{2k+1}^+ C_{2k+1}^+ b_{2k+1}^-\right]\Psi(t), \tag{23}$$

where coefficients C_l depend on the initial state and come from a sum of all possible permutations of combinations of creation-annihilation operators ($a_- a_-^\dagger$, $a_-^\dagger a_-$, $a_+^\dagger a_+$, $a_+ a_+^\dagger$ for 2 virtual photons involved, $a_+ a_-^\dagger a_- a_+^\dagger$,$a_+^\dagger a_- a_-^\dagger a_+$ and two more terms for 4 virtual photons involved and so on), which do not change neither the occupation nor the frequency of photonic states. Assuming that $n \gg 2k$ and taking into account the relations $a\,|n\rangle = n\,|n\rangle$, $a^\dagger\,|n\rangle \approx n\,|n\rangle$ we arrive at

$$C_l \approx \frac{1}{(\sqrt{n})^l}\sum_{m=0}^{\infty}\frac{(-1)^{j+m}(\eta\sqrt{n})^{l+2m}}{(l+2m)!}\frac{(l+2m)!}{m!(l+m)!} = \frac{(-1)^j}{(\sqrt{n})^l}J_l(\eta\sqrt{n}), \tag{24}$$

where $j = \mod(l, 2)$, J_l is the Bessel function of the first kind.

If the initial state $\Psi = |\beta, \alpha, \alpha\rangle$, where α is a real number, Supplementary Eq. (23), is simplified to

$$\sum_{k=-\infty}^{\infty} \left[\frac{(-1)^k}{\alpha^{2k}} J_{2k}(\theta) \Big(\hat{A}^{+-}_{2k} b^{-}_{-k} b^{+}_{k} + \hat{A}^{-+}_{2k} b^{+}_{-k} b^{-}_{k} \Big) + \frac{(-1)^k}{\alpha^{2k+1}} J_{2k+1}(\theta) \Big(\hat{A}^{+}_{2k+1} b^{-}_{2k+1} - \hat{A}^{-}_{2k+1} b^{+}_{2k+1} \Big) \right] \Psi \tag{25}$$

and in case of $\Psi = |0, \alpha, \alpha\rangle$

$$U\Psi \approx \sum_{k=-\infty}^{\infty} \left[\frac{(-1)^k}{\alpha^{2k}} J_{2k}(\theta) \hat{A}^{+-}_{2k} |0\rangle_{2k} \otimes |\alpha, \alpha\rangle + \frac{(-1)^k}{\alpha^{2k+1}} J_{2k+1}(\theta) \hat{A}^{-}_{2k+1} |1\rangle_{2k+1} \otimes |\alpha, \alpha\rangle \right], \tag{26}$$

where $\theta = 2\eta\alpha$. Taking into account that $b^{+}_{2k+1} = |1\rangle_{2(k+p)+1}\langle 0|_{2p}$, we can directly write an expression for the expectation value of the single-photon creation operator at ω_{2k+1}

$$\langle b^{+}_{2k+1} \rangle = \sum_{p=-\infty}^{\infty} \frac{(-1)^{k+p+p}}{\alpha^{2(k+p)+1}} J_{2(k+p)+1}(\theta) J_{2p}(\theta) \langle \hat{A}^{-}_{2(k+p)+1} \hat{A}^{+-}_{-2p} \rangle. \tag{27}$$

Usind standard textbook formulas for Bessel functions, it is simplified to

$$\langle b^{+}_{2k+1} \rangle = \frac{(-1)^k J_{2k+1}(2\theta)}{2\alpha^{2k+1}} \langle \hat{A}^{-}_{2k+1} \rangle. \tag{28}$$

Here we use the following property: $\alpha^{-(2(k+p)+1)} \langle \hat{A}^{-}_{2(k+p)+1} \hat{A}^{+-}_{-2p} \rangle \approx \alpha^{-(2k+1)} \langle \hat{A}^{-}_{2k+1} \rangle$ for $\alpha \gg 1$. Taking into account that $\langle \hat{A}^{-}_{2k+1} \rangle \approx \alpha^{2k+1}$, we simplify the expression to

$$\langle b^{+}_{2k+1} \rangle = \frac{(-1)^k}{2} J_{2k+1}(2\Omega\Delta t), \tag{29}$$

where $\Omega\Delta t = \theta$. The answer is exactly as in Supplementary Eq. (16). The coherent emission amplitude at each mode is

$$V_{2k+1} = \frac{\hbar\Gamma_1}{\mu} \langle b_{2k+1} \rangle, \tag{30}$$

and power

$$W_{2k+1} = \frac{V^2_{2k+1}}{2Z_0}, \tag{31}$$

where Z_0 is the line impedance, we calculate the coherent wave energy at each cycle, substituting Supplementary Eq. (29) into Supplementary Eq. (20) and integrating over time t. Taking into account that $\Gamma_1 = \frac{\hbar\omega\mu^2 Z_0}{\hbar^2}$ and $\int_0^\infty \langle b_{2k+1} \rangle^2 dt = \int_0^\infty e^{-\Gamma_1 t} = \Gamma_1^{-1}$, we find the generated photon number in two directions to be

$$\frac{E_{\pm(2k+1)}}{\hbar\omega} = \frac{J^2_{\pm(2k+1)}(2\Omega\Delta t)}{4}. \tag{32}$$

Although the analytical solution has been found for the approximated case of strong drive, it can be generalised for arbitrary initial driving states as

$$\langle b^{+}_{2k+1} \rangle = D^{-}_{2k+1} \langle \hat{A}^{-}_{2k+1} \rangle, \tag{33}$$

where D^{-}_{2k+1} is a coefficient dependent on the driving amplitudes. This means that only states at frequency $\omega_0 \pm (2k+1)\delta\omega$ with odd indexes $2k+1 > 0$ can be created. Creation of the single-photon state, requires annihilation of $k+1$ photons at at ω_+ and creation of k photons at ω_-.

Supplementary Note 3. Quantum mixing

Now we will consider an evolution from two sequential pulses with frequencies ω_- and ω_+. The first pulse results in the atomic excitation described by the single-photon field $|\beta\rangle_-$, which comes from process $|\beta_-, \alpha, \alpha\rangle = B_-(1 +$

$\beta_-\frac{b_-^+ a_-}{\alpha_-})|0,\alpha_-,\alpha_+\rangle$, where $B_- = \sqrt{1+\beta_-^2}$. The state is then interacts with coherent state $|\alpha\rangle_+$ of the second pulse. Among all processes of the third and higher orders, the only non-trivial one, leading to creation of the new frequency mode, is described by $A_3^- b_3^+ = a_+ a_-^\dagger a_+ b_3^+$ because at most one photon is emitted from $|\beta\rangle_-$ at ω_-. All other processes are prohibited due to lack of photons at ω_-. It results in creation of the single-photon state at $2\omega_+ - \omega_- = \omega_0 + 3\delta\omega$.

The evolution is described as

$$U(t', t'')|0,\alpha_-,\alpha_+\rangle \approx B_+\left(1+\beta_+\frac{a_+b_+^+ - a_+^\dagger b_+^-}{\sqrt{a_+^\dagger a+}}\right) \times B_-\left(1+\beta_-\frac{a_-b_-^+}{\sqrt{a_-^\dagger a_-}}\right)|0,\alpha_-,\alpha_+\rangle, \tag{34}$$

where $\beta_\pm = \tan(\theta_\pm/2)$, $B_\pm = \sqrt{1+\beta_\pm^2}$, $\theta_- = 2g_-\alpha_-(t'-t)$, $\theta_+ = 2g_-\alpha_-(t''-t')$. The equation is simplified to

$$U(t', t'')|0,\alpha_-,\alpha_+\rangle \approx B_+B_-\left(1 - \hat{\beta}_+^-\hat{\beta}_-^+ + \hat{\beta}_+^+ + \hat{\beta}_-^+\right)|0,\alpha_-,\alpha_+\rangle, \tag{35}$$

where operators $\hat{\beta}_\pm^+ = \beta_\pm a_\pm b_\pm^+ (a_\pm^\dagger a_\pm)^{-1/2}$ and $\hat{\beta}_\pm^- = (\hat{\beta}_\pm^+)^\dagger$. Among all possible operators of Supplementary Eq. (22) of orders higher or equal to 3, only $A_3^- b_3^+$ creates the new frequency field because only

$$\langle A_3^- b_3^+\rangle = B_+^2 B_- \langle 0,\alpha_-,\alpha_+|\hat{\beta}_+^- a_+ a_-^\dagger a_+ b_3^+ \hat{\beta}_+^- \hat{\beta}_-^+|0,\alpha_-,\alpha_+\rangle \tag{36}$$

is non-zero. And we find

$$\langle b_3^+\rangle = B_+^2 B_- \beta_+^2 \beta_-. \tag{37}$$

Finally, the field amplitude is found to be

$$\langle b_3^+\rangle \approx \sin^2[\Omega_+(t''-t')]\sin[\Omega_-(t'-t)], \tag{38}$$

where $\Omega_\pm = g\alpha_\pm$.

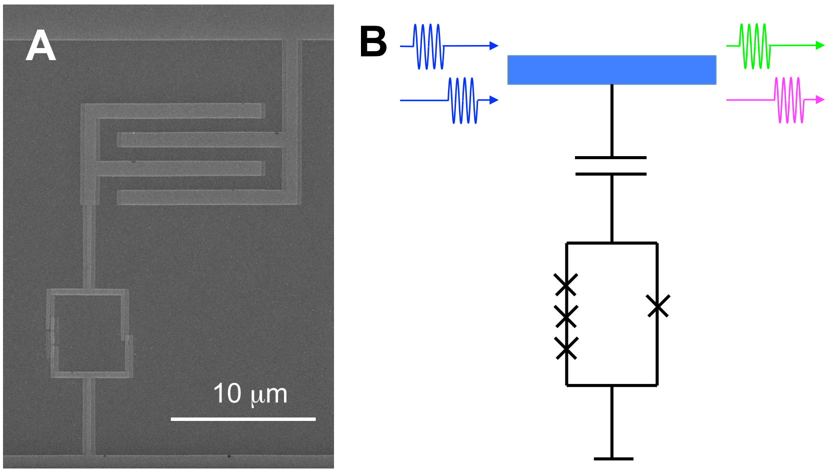

Supplementary Figure 1: **The device**. (**A**) An SEM image of an artificial atom coupled through an interdigitated capacitance to a transmission line. (**B**) A schematic representation of the device. The device is a superconducting loop with four Josephson junctions.

Supplementary Note 4. Two-photon quantum mixing

In case of an equally spaced three-level system described by $|0\rangle$, $|1\rangle$ and $|2\rangle$, with transition energies $\hbar\omega_{10} = \hbar\omega_{21}$, the resonance drive results in excitation of the system, leading to state $\Psi = C(|0\rangle + \gamma_1|1\rangle + |\gamma_2|^2\rangle)$, where $C =$

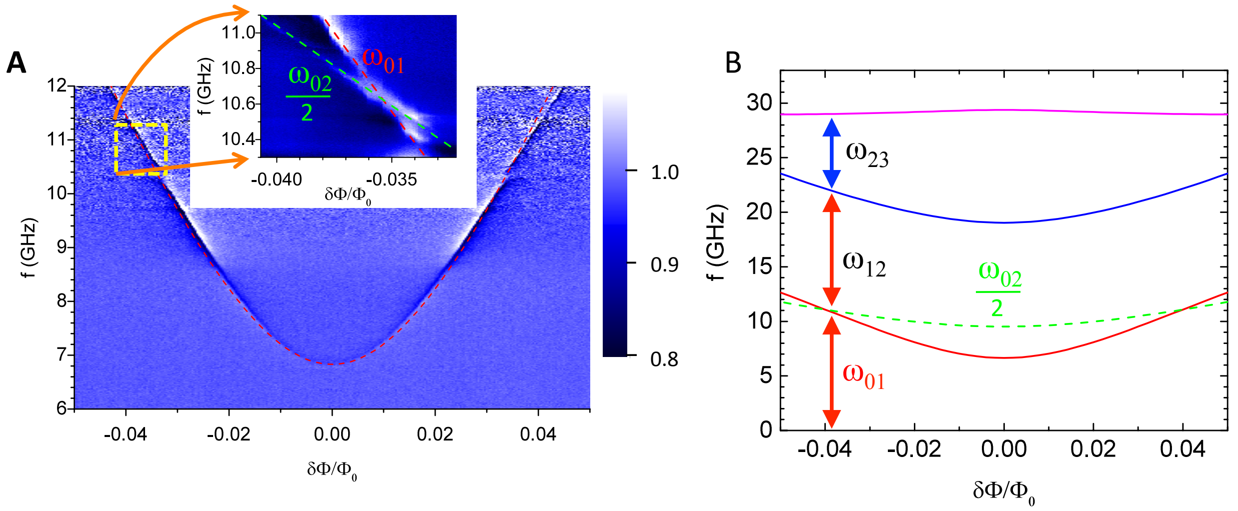

Supplementary Figure 2: **The spectroscopy.** (**A**) A transmission spectroscopy of the system as a function of the normalised magnetic flux bias measured by a vector network analyser. The red dashed curve shows calculations of the energy $\hbar\omega_{01}$. An inset shows the transmission phase under a strong drive. A transition line at ω_{01} crosses a line from the two-photon transition process at ω_{02}. The crossing takes place at a point where $\omega_{01} = \omega_{12}$. (**B**) A simulation of the system energies with parameters close to our artificial atom. The red arrowed lines show two equal transition frequencies $\omega_{01} = \omega_{12} \neq \omega_{23}$.

$\left[1 + |\gamma_1|^2 + |\gamma_2|^2\right]^{-\frac{1}{2}}$. We introduce two-state creation/annihilation operators $c^\dagger$, c with the following properties: $c^\dagger|0\rangle = |1\rangle$, $c^\dagger|1\rangle = |2\rangle$, $c^\dagger|2\rangle = 0$, $c|2\rangle = |1\rangle$, $c|1\rangle = |0\rangle$, $c|0\rangle = 0$. In the case of the same pulse sequence the first pulse applied at ω_- during time $[t', t]$ is followed by another pulse ω_+ during $[t'', t']$.

Similarly to Supplementary Eq. (34), the evolution is presented as

$$U(t'', t) = C_+\left[1 + \hat{\gamma}_+^\dagger + \hat{\gamma}_+ + \hat{\gamma}_{2+}^\dagger + \hat{\gamma}_{2+}\right] \times C_-\left[1 + \hat{\gamma}_-^\dagger + \hat{\gamma}_{2-}^\dagger\right], \tag{39}$$

where $\hat{\gamma}^\dagger_{1\pm} = \gamma^+_{1\pm} a_\pm c^\dagger_\pm$, $\hat{\gamma}^+_{1\pm} = \gamma^-_{1\pm} a^\dagger_\pm c_\pm$, $\hat{\gamma}^\dagger_{2\pm} = \gamma^+_{2\pm}(a_\pm c^\dagger_\pm)^2$, $\hat{\gamma}^+_{2\pm} = \gamma^-_{2\pm}(a^\dagger_\pm c_\pm)^2$.

$$U(t'', t) = C_+C_-\left[1 + \hat{\gamma}_+^\dagger + \hat{\gamma}_+ + \hat{\gamma}_-^\dagger + \hat{\gamma}_{2+}^\dagger + \hat{\gamma}_{2+} + \hat{\gamma}_{2-}^\dagger + \hat{\gamma}_+^\dagger\hat{\gamma}_-^\dagger + \hat{\gamma}_+\hat{\gamma}_-^\dagger + \hat{\gamma}_+\hat{\gamma}_{2-}^\dagger + \hat{\gamma}_{2+}\hat{\gamma}_{2-}^\dagger\right]. \tag{40}$$

As an example, some terms contributing into the peaks with additional to the single-photon wave-mixing are

$$3\omega_+ - 2\omega_- : \quad \langle\hat{\gamma}_+^\dagger A_5^- c_5^+(\hat{\gamma}_{2+}\hat{\gamma}_{2-}^\dagger)^\dagger\rangle = \langle\hat{\gamma}_+^\dagger A_5^- c_5^+\hat{\gamma}_{2-}\hat{\gamma}_{2+}^\dagger\rangle \quad \to \langle a_+a_-^\dagger a_-^\dagger a_+a_+\rangle \tag{41a}$$

$$2\omega_- - \omega_+ : \quad \langle\hat{\gamma}_{2-}^\dagger A_3^- c_{-3}^+(\hat{\gamma}_+)^\dagger\rangle \quad \to \langle a_-a_-a_+^\dagger\rangle. \tag{41b}$$

Supplementary Methods. The device

We have studied the quantum mixing on an artificial atom based on a superconducting qubit geometry shown in Supplementary Fig. 1. The atom is a micron-size loop with four nanometer-scale Josephson junctions fabricated by techniques of electron beam litography and shadow two-angle evaporation. The atom is capacitively coupled to a transmission line. The energy splitting controlled by external magnetic field is approximated as

$$\hbar\omega_a = \sqrt{2I_p\delta\Phi^2 + \Delta^2}, \tag{42}$$

where persistent current $I_p = 40$ nA, $\delta\Phi$ is a magnetic flux threading through the loop and $\Delta = \hbar\times$ 6.8 GHz is the tunnelling energy. The atom is strongly coupled to the line with negligible dephasing, comparing to photon emission rate $\Gamma_1/2\pi = 20$ MHz.

We apply coherent microwaves to drive the atom and detect the signal by a spectrum analyser after amplifying it by a cryogenic and a room-temperature amplifiers. A transmission spectroscopy ([1, 2]) is shown in Supplementary Fig. 2A. At magnetic flux bias $\delta\Phi/\Phi_0 \approx \pm\, 0.035$ transition frequency between the ground and excited state are equal. Therefore the resonant microwave results in population of both levels, which is equivalent to storage of the two-photon state $|\gamma\rangle$.

Supplementary References

[1] O. Astafiev *et al.* Resonance fluorescence of a single artificial atom, *Science* **327**, pp. 840-843, (2010).
[2] A. A. Abdumalikov Jr *et al.* Dynamics of coherent and incoherent emission from an artificial atom in a 1D space, *Phys. Rev. Lett.* **107**, 043604, (2011).
